# THE DEVELOPMENT AND UTILIZATION OF IMAGE:NYC: THE INTERACTIVE MAP OF AGING

**DOI:** 10.1093/geroni/igad104.0711

**Published:** 2023-12-21

**Authors:** Elana Kieffer, Clara Scher

**Affiliations:** The New York Academy of Medicine, New York City, New York, United States; Rutgers, The State University of New Jersey, New Brunswick, New Jersey, United States

## Abstract

Online interactive maps are an effective tool to visualize and understand socio-spatial locations of populations layered across the availability of relevant resources. These maps are integral in efforts to advance policies, programs and planning for specific sociodemographic groups. This presentation will describe the development and impact of IMAGE:NYC, The Interactive Map of Aging by The New York Academy of Medicine (NYAM) in partnership with the Center for Urban Research at The Graduate Center at the City University of New York. IMAGE:NYC displays the current and projected population of adults aged 65 and older in New York City with overlays of age-friendly resources, services, and amenities at the neighborhood level. The map draws on several data sources such as the 2016-2020 American Community Survey, local and federal voting patterns, and up-to-date information on health, recreational and housing services. The map has served as an invaluable tool for NYC policymakers, planners, researchers, health care and social service providers. In addition, the IMAGE:NYC model has also been replicated by Rutgers University to create similar maps for two counties in New Jersey. This session will demonstrate the utility of this mapping tool and concrete ways to develop a similar map in other localities.

